# Identification of Components of the Sex Pheromone of the kōwhai Moth, Uresiphita Polygonalis Maorialis, a New Zealand Native Crambid

**DOI:** 10.1007/s10886-025-01564-8

**Published:** 2025-01-22

**Authors:** Ashraf M. El-Sayed, A. R. Gibb

**Affiliations:** 1 Canterbury Research Centre, The New Zealand Institute for Plant and Food Research Limited, Lincoln, 8152 New Zealand; 2 46 Hay Street, Bromley, Christchurch, 8062 New Zealand

**Keywords:** *Uresiphita polygonalis maorialis*, Lepidoptera, Crambidae, Sex pheromones, Tetradecyl acetate, (*E*)-11-tetradecenyl acetate, (*Z*)-11-tetradecenyl acetate, (*Z*)-11-hexadecenyl acetate

## Abstract

The identification of sex pheromones in native New Zealand moths has been limited, largely due to their minimal pest impact on agricultural ecosystems. The kōwhai moth, *Uresiphita polygonalis maorialis*, a native crambid, is known for its herbivory on *Sophora* spp. and *Lupinus arboreus* leaves. Understanding the chemical ecology of this species is essential for studying its behavior, population dynamics, and ecological interactions. In this study, the female sex pheromone of *U. polygonalis maorialis* was analyzed using coupled gas chromatography-electroantennogram detection (GC-EAD). This approach identified four antennally active compounds in the female gland extracts. Subsequent gas chromatography-mass spectrometry (GC-MS) and chemical derivatization revealed these compounds to be tetradecyl acetate (14:Ac), (E)-11-tetradecenyl acetate (E11-14:Ac), (Z)-11-tetradecenyl acetate (Z11-14:Ac), and (Z)-11-hexadecenyl acetate (Z11-16:Ac). Field trapping experiments evaluated various combinations of these four EAD-active compounds and (E)-11-hexadecenyl acetate (E11-16:Ac). Results indicated that traps baited with blends containing E11-14:Ac, Z11-14:Ac, and Z11-16:Ac captured significantly more males compared to unbaited delta traps. A blend ratio of 144:84:72 µg (E11-14:Ac: Z11-14:Ac: Z11-16:Ac) proved the most effective, capturing the highest number of males. Male captures were recorded from late November to late February, peaking in late December, suggesting a univoltine population in Canterbury. Among the three tested doses, the 300 µg and 1000 µg doses of the three-component blend were the most effective. The identification of the sex pheromone components of *U. polygonalis maorialis* provides a valuable tool for monitoring this species, contributing to a deeper understanding of its population densities and distribution within its native range. It also offers insights into the evolutionary development of pheromone communication within the genus, shedding light on species divergence and adaptation.

**Supplementary Information:**

The online version contains supplementary material available at 10.1007/s10886-025-01564-8.

## Introduction

The kōwhai moth, *Uresiphita polygonalis maorialis*, holds a prominent place in New Zealand’s diverse ecosystems, primarily feeding on the leaves of kōwhai (*Sophora* spp.) and tree lupine, *Lupinus arboreus* (Field et al. 2000). In New Zealand, this species is closely associated with the kōwhai tree, *Sophora microphylla*, which produces striking golden yellow flowers that create a stunning display in early spring. This native plant is highly valued in Māori culture, not only for its beauty but also for its medicinal properties and significance in traditional practices. Despite its iconic presence, much of this insect’s biology remains shrouded in mystery. While it holds a prominent place in local culture, no previous efforts have been made to identify its sex pheromone, leaving a significant gap in our understanding of its behavioral ecology.The discovery of the sex pheromone of *U. polygonalis maorialis* will offer valuable insights into the evolutionary development of pheromone communication within the genus *Uresiphita*. Understanding pheromone evolution in this genus can shed light on how chemical communication has influenced species divergence and adaptation. This knowledge enhances our understanding of the role chemical signals play in shaping reproductive isolation, speciation, and the ecological relationships within diverse ecosystems.

The identification of sex pheromones for native New Zealand moths has been relatively limited, largely due to the fact that only a handful of indigenous moth species are regarded as pests within our agricultural ecosystems (Dugdale 1990; Dymock and Crowe 2012). This unique ecological context, characterized by a relatively low number of economically significant moth pests, has resulted in a corresponding scarcity of research focused on moth pheromones in New Zealand. However, the importance of understanding the chemical communication systems of native moth species extends beyond their agricultural impact. These species often play vital roles in local ecosystems and may have cultural or conservation significance. Therefore, efforts to identify their sex pheromones hold promise for enhancing our understanding of their reproductive biology, population dynamics, and ecological interactions, contributing to both scientific knowledge and conservation efforts in New Zealand’s diverse natural landscapes.

The objective of this study is to identify the sex pheromone of the kōwhai moth, *Uresiphita polygonalis maorialis*, a species cherished for its iconic status rather than its agricultural pest status. While not considered a significant agricultural pest, understanding the chemical communication system of this iconic species holds profound ecological implications. By elucidating the sex pheromone of *U. polygonalis maorialis*, we can gain insights into its mating behavior, population dynamics, and habitat requirements. To achieve this objective, we have employed a combination of chemical ecology techniques, including gas chromatography-mass spectrometry (GC-MS) to isolate and identify candidate pheromone compounds from female-produced pheromone glands. Behavioral bioassays have been conducted to assess the attractiveness of synthetic pheromone blends to male moths in field trapping trials.

## Methods and Materials

### Insects

Larvae of *U. polygonalis maorialis* were collected from kōwhai tree leaves in the Department of Conservation (DOC)-administered Lyttelton Reserve, Canterbury during the spring and summer (October/November 2019 and 2020). All larvae were allowed to feed on leaves until pupation. Pupae were housed individually in 30-ml plastic cups (Solo, Mason, MI) with a moist dental roll. All pupae were kept in a controlled environment room under a 16 h:8 h L: D photoperiod and 23 °C, and newly emerged adults were sexed under the microscope. Virgin females were used in sex pheromone extraction.

### Pheromone Gland Extracts

Pheromone gland extracts were taken from 24 to 72 h old actively calling females kept in a 40 cm^3^ humidified plastic container in the quarantine facilities at 2–3 h into scotophase. Excised pheromone glands were eluted for 10 min in analytical grade n-hexane and the supernatant was collected and stored at -80^○^C, before being used for GC-MS and GC-EAD analyses. Quantification of the amount of each compound in the female sex pheromone gland was conducted using the external standard method (Scott 1995). In this method, known amounts (0.1, 1.0, 10.0, and 100.0 ng) of each compound were injected into the GC-MS. The results were subjected to a linear regression analysis (SAS Institute Inc. 1998) to determine the relationship between the amount of compound injected and the peak area of the chromatogram.

### Chemicals

Tetradecyl acetate (14:Ac), (E)-11-tetradecenyl acetate (E11-14:Ac), (Z)-11-tetradecenyl acetate (Z11-14:Ac), (E)-11-hexadecenyl acetate (E11-16:Ac), and (Z)-11-hexadecenyl acetate (Z11-16:Ac), were purchased from Pherobank, Wageningen, The Netherlands, and were > 97% isomerically pure by GC analysis. Dimethyl disulfide (DMDS) was obtained from Merck, Darmstadt, Germany and n-hexane (99%), iodine, sodium sulphate and sodium thiosulphate from BDH Laboratory Supplies, Poole, England.

### Dimethyldisulfide (DMDS) Derivatization

Dimethyl disulfide (DMDS) derivatization was used to identify the position of the double bond in the pheromone compounds. DMDS derivatizations of the synthetic E11-14:Ac, Z11-14:Ac, Z11-16:Ac (10 ng in 20 µl of n-hexane) and 15 female equivalent (FE) gland extracts of *U. polygonalis maorialis* were carried out following the methods of Buser et al. (1983). Approximately 50 µL of DMDS and 5 µL of iodine solution (60 mg of I_2_ in 1 mL of diethyl ether) were added to the solution of interest in a 1.8 mL glass vial. The vial was sealed with a Teflon-lined cap and the reaction was allowed to proceed at 40 °C for 15 h. To stop the reaction, 50 µL of a 5% aqueous solution of sodium thiosulfate was added. The organic layer was then dried using anhydrous sodium sulphate, and subsequently filtered using a small glass pipette containing a plug of cotton wool to remove any remaining solid particles. The filtered solution was concentrated by blowing it down with a stream of argon to < 5 µL. A 1 µL aliquot of this concentrated solution was immediately analyzed by GC-MS.

### Gas chromatography-electroantennographic Detection (GC-EAD)

To determine the antennally active components in the pheromone glands of *U. polygonalis maorialis*, pheromone gland extracts were analyzed by GC-EAD using a Varian 3800 gas chromatograph equipped with both polar and non-polar columns, coupled to an EAD Recording Unit. Electroantennographic recordings were conducted by excising the distal part of the antenna, which was placed between two glass capillaries filled with Ringer’s solution and silver electrodes. The base of the antennal preparation was grounded, while the tip was connected to a Syntech AC/DC probe, linked to a Syntech IDAC-2 digital converter (Syntech, Kirchzarten, Germany). The acquired data were analyzed using Syntech GC-EAD 2011 software (version 1.2.3, Ockenfels Syntech GmbH). We extracted the sex pheromone glands from 60 female *U. polygonalis maorialis* using 50 µL of hexane. After removing the glands, the solvent was concentrated down to approximately 3 µL. From this concentrated solution, 1 µL, containing 20 female equivalents (FE), was injected for analysis. Extracts were first run on a VF-5ms (Agilent Technologies, California, USA) capillary column (30 m x 0.25 mm ID x 0.5 μm film thickness) and then a polar BPX-70 capillary column (SGE, Melbourne, Australia) (30 m x 0.25 mm ID x 0.25 μm film thickness). Both capillary columns had 1:1 split outlets achieved using a fused silica press-fit Y-splitter (Alltech, Deerfield, IL, USA). Helium was used as the carrier gas at a flow rate of 1 ml/min and injections were in splitless mode. The injector temperature was set at 220 °C, the flame ionization detector at 300 °C and the GC oven temperature was programmed from 80 °C (1 min hold) to 240 °C at 10 °C/min and held for 30 min. An excised male *U. polygonalis maorialis* antenna was positioned between two glass electrodes, containing Beadle-Ephrussi Ringer’s solution (Ephrussi and Beadle 1936) with 10% polyvinylpyrrolidone (Molecular Weight 360,000) (Sigma Chemical Co., N.S.W., Australia). Each glass electrode held a piece of 1 mm width silver wire that electrically connected the preparation to the recording preamplification unit. The EAD exit port temperature was maintained at 200 °C and the antennal preparation was placed in a charcoal-filtered and humidified 400 ml/min airstream. Kováts retention indexes (KI’s) were calculated for the antennally active compounds and the standards using the Kováts Calculator and Match Search (El-Sayed 2024), on both the polar and non-polar GC column.

### Gas Chromatography-Mass Spectrometry (GC-MS)

Pheromone gland extracts of *U. polygonalis maorialis* and 14:Ac, E11-14:Ac, E11-16:Ac, and Z11-16:Ac standards were analyzed by GC-MS on a VF-5ms capillary column (30 m x 0.25 mm ID x 0.25 *µ*m film thickness) (Varian Inc., California, USA) and then on a polar BPX70 capillary column (SGE, Melbourne, Australia) (30 m x 0.25 mm ID x 0.25 μm film thickness), using a Varian 3800 gas chromatograph in splitless injection mode coupled to a Varian 2200 MS. For the VF-5ms column the injector temperature was set at 220 °C and the GC oven temperature programmed from 80 °C (1 min hold) to 240 °C at 10 °C/min and held for 15 min. For analysis of pheromone gland extract using the BPX-70 column, the temperature program was as follows: GC oven temperature programmed from 80 °C (1 min hold) to 130 °C at 10 °C/min and held for 3 min, then ramped to 190 °C at 5 °C/min and then held for 20 min.

DMDS derivatizations of a *U. polygonalis maorialis* 15FE pheromone gland extracts, synthetic E11-14:Ac, Z11-14:Ac, and Z11-16:Ac standards were analyzed by GC-MS on the VF-5ms capillary column The injector temperature was set at 250 °C and the GC oven temperature programmed from 80 °C (1 min hold) to 240 °C at 10 °C/min and held for 15 min. After derivatization with DMDS, the resulting isomeric adducts for the synthetic standards, while having identical spectra, were compared with any adducts formed in the derivatized 15FE gland extract of *U. polygonalis maorialis.*

### Field Trapping Trials

In the two field trials conducted in this study, green delta traps (Clare et al. 2000) obtained from Etec Crop Solutions Ltd in Auckland, New Zealand, were suspended at a height between 1 and 1.5 m on kōwhai tree branches in the DOC-administered Lyttelton Reserve, Canterbury, New Zealand (coordinates: 43°35’55.8"S 172°44’08.6"E). The traps were deployed in a randomized block design, with five trap lines established at approximately 20-30-m intervals. Within each trap line, seven trapping stations were randomly assigned at approximately 15–20 m intervals for Trial 1, while four trapping stations were assigned for Trial 2. To minimize positional bias, the traps were rotated to different trapping stations after each check.

### Testing Blends of Candidate Pheromone Compounds

In this trial, six pheromone blends were prepared at the loading of 300 µg in 200 µl of n-hexane and added to the large ‘wells’ of red rubber septa (West Pharmaceutical Services, Kearney, NE, USA). The solvent was allowed to evaporate in a fume hood, and the septa were stored in heat-sealed foil bags at − 20 °C until use. These blends included the following: 1- a two-component blend (E11-14:Ac, and Z11-14:Ac, in amounts of 190, and 110 µg, respectively); 2- a three-component blend (14:Ac, E11-14:Ac, and Z11-14:Ac, in amounts of 55, 155, and 90 µg, respectively); 3- a four-component blend (14:Ac, E11-14:Ac, Z11-14:Ac, and Z11-16:Ac, in amounts of 45, 125, 70, and 60 µg, respectively); 4- a five-component blend (14:Ac, E11-14:Ac, Z11-14:Ac, E11-16:Ac, and Z11-16:Ac, in amounts of 45, 125, 70, 3, and 57 µg, respectively); 5- a three-component blend- (E11-14:Ac, Z11-14:Ac, and Z11-16:Ac, in amounts of 144, 84, and 72 µg, respectively); 6- a four-component blend (E11-14:Ac, Z11-14:Ac, E11-16:Ac, and Z11-16:Ac, in amounts of 144, 84, 3, and 69 µg, respectively). The trial was deployed for 15 weeks, from 15 November 2019 to February 28, 2020. The lures were replaced at five-week intervals.

### Testing Three Doses of the three-component Blend

In this trial, three doses (100, 300, and 1000 µg in 200 µl of n-hexane) of the three-component blend were tested in the same location as the previous trial for 5 weeks from 7 December 2020 to January 11, 2021. The three doses tested were as follow: 1- (E11-14:Ac, Z11-14:Ac, and Z11-16:Ac, in amounts of 48, 28, and 24 µg, respectively); 2- (E11-14:Ac, Z11-14:Ac, and Z11-16:Ac, in amounts of 144, 84, and 72 µg, respectively); 3-(E11-14:Ac, Z11-14:Ac, and Z11-16:Ac, in amounts of 480, 280, and 240 µg, respectively).

In the two trials, the pheromone-impregnated septa were placed on the sticky panel of the trap. Traps baited solely with red rubber septa impregnated with 200 µl of n-hexane served as negative controls. Five replicates for each treatment were tested. Traps were checked weekly, and moths captured were taken to the laboratory for identification. The total number of males captured in each replicate within each treatment over the trapping period was combined for statistical analysis.

### Data Analysis

The effect of pheromone treatment on the mean number of males *U. polygonalis maorialis* captured was tested using ANOVA (SAS Institute Inc. 1998) after variances were stabilized using the √x + 1 transformation. Significantly different treatment means were identified by post hoc using the Tukey-Kramer Test at the 5% level (SAS Institute Inc. 1998). Zero values were not included in the ANOVA or Tukey-Kramer Test analysis.

## Results

### Gas chromatography-electroantennographic Detection (GC-EAD)

**Fig. 1:**
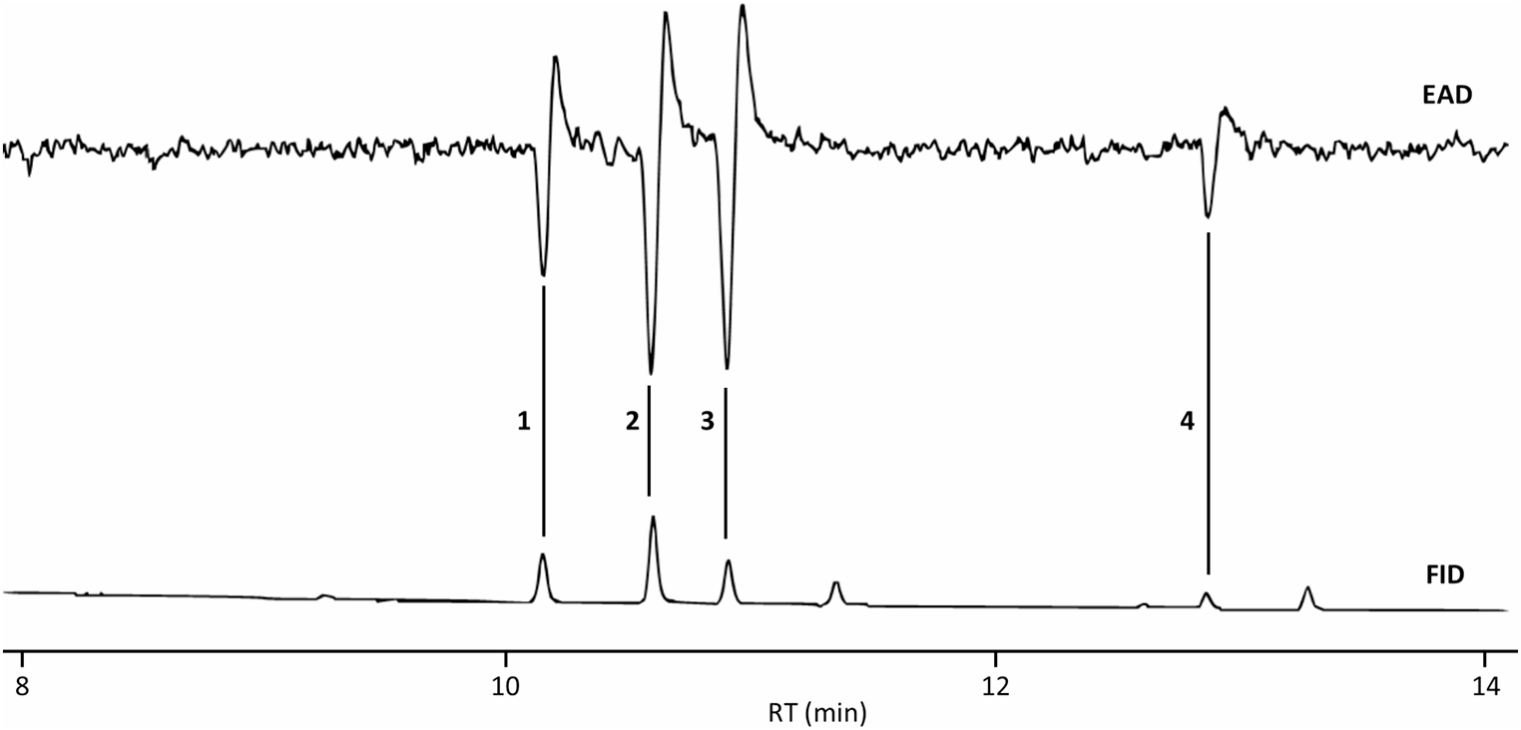
Coupled GC/EAD responses of an antenna of 4-day-old male *Uresiphita polygonalis maorialis* to female gland extract (20FE). Chromatographic column and conditions: A polar BPX70 capillary column was used for the analyses, and the oven temperature was programmed from 80 °C (held for 1 min) to 240 °C at 10 °C/min. 1 = 14:Ac, 2 = E11-14:Ac, 3 = Z11-14:Ac, and 4 = Z14-16:Ac

Analysis of the female sex pheromone gland extracts by GC-EAD unveiled four compounds that triggered notable EAD responses from male moth antennae (Fig. 1). These compounds were subsequently identified as 14:Ac (1), E11-14:Ac (2), Z11-14:Ac (3), and Z11-16:Ac (4). Notably, E11-14:Ac and Z11-14:Ac exhibited the most pronounced EAD responses, suggesting their potential significance as primary components of the kōwhai moth’s sex pheromone blend. Conversely, both 14:Ac and Z11-16:Ac elicited smaller but comparable EAD responses.

### Chemical Identification

All four compounds produced an ion fragment with m/z 61 (CH3COOH2^+^), characteristic of straight-chain acetates. Compound 1 exhibited an ion fragment of *m/z* 196 (M^+^- 60), while compounds 2 and 3 displayed an ion fragment of *m/z* 194 (M^+^-60). Similarly, compound 4 yielded an ion fragment of m/z 222 (M^+^- 60). This observation indicates that compound 1 corresponds to a saturated 14-carbon acetate (14:Ac), while compounds 2 and 3 are likely monounsaturated 14-carbon acetates. Similarly, compound 4 is presumed to be a monounsaturated 16-carbon acetate.

The similarity in retention indices (RI) between compounds 2 and 3, coupled with the comparison of their RI values with each of the double-bond positional isomers of tetradecenyl acetates reported by El-Sayed in 2024, strongly suggested that both compounds 2 and 3 are Δ11–14 isomers. The comparison of the GC retention times (on BPX 70 and VF-5ms columns, Table 1) of compounds 1, 2, and 3 with those of authentic 14:Ac, E11-14:Ac, and Z11-14:Ac enabled us to unambiguously conclude that compound 1 corresponds to 14:Ac, compound 2 to E11-14:Ac, and compound 3 to Z11-14:Ac, respectively. Finally, the comparison of the RI value of compound 4 with each of the double-bond positional isomers of hexadecenyl acetates reported by El-Sayed in 2024 strongly suggested that compound 4 is a Δ11–16 isomer. The comparison of the GC retention times (on BPX 70 and VF-5ms columns, Table 1) of compound 4 with those of authentic Z11-16:Ac enabled us to conclude that compound 4 corresponds to Z11-16:Ac. Additionally, a trace peak eluted just before compound 4 exhibited very similar ion fragments to compound 4, suggesting the potential geometrical isomer E11-16:Ac.

**Table 1:** Kovats retention index values for pheromone components in the extracts and synthetic standards on BPX 70 and VF-5ms columns

Compound	Kovats Retention Index			
	Extracts (BPX 70)	Synthetic (BPX 70)	Extracts (VF-5ms)	Synthetic (VF-5ms)
14:Ac	2177	2177	1810	1810
E11-14:Ac	2209	2209	1803	1803
Z11-14:Ac	2225	2225	1808	1808
E11-16:Ac	2422	2422	1996	1996
Z11-16:Ac	2444	2444	1998	1998

Double-bond positions in the monounsaturated 14- and 16-carbon acetate of the *U. polygonalis maorialis* pheromone extract were further confirmed by reaction with DMDS. GC-MS analysis of the resulting mixture showed the presence of the DMDS adduct of Δ11-tetradecenyl acetate [m/z 348 (M^+^) and major fragments at m/z 259 and 89] and Δ11-hexadecenyl acetate [m/z 376 (M^+^) and major fragments at *m/z* 259 and 117].

**Fig. 2:**
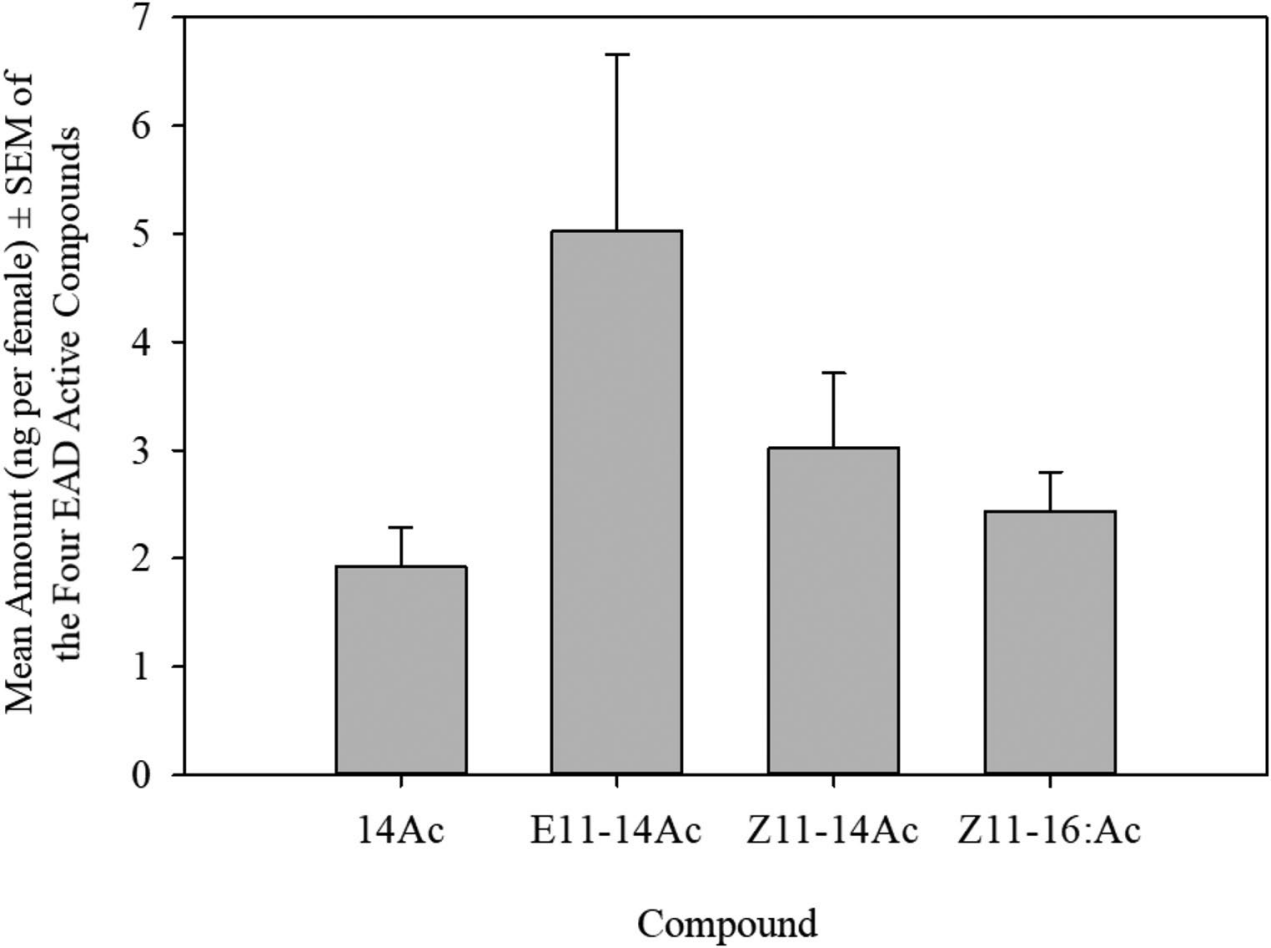
Mean amount (ng per female) ± SEM of the four pheromone gland compounds in *Uresiphita polygonalis maorialis* (*N* = 25)

The amount of each of the four pheromone compounds per female is given in Fig. 2. The female sex pheromone gland of *U. polygonalis maorialis*, contains an average of 5.1 ng of E11-14:Ac, 3.2 ng of Z11-14:Ac, 2.4 ng of Z11-16:Ac, and 1.9 ng of 14:Ac per female.

### Field Trapping Experiments

#### Testing Blends of Candidate Pheromone Compounds

**Fig. 3:**
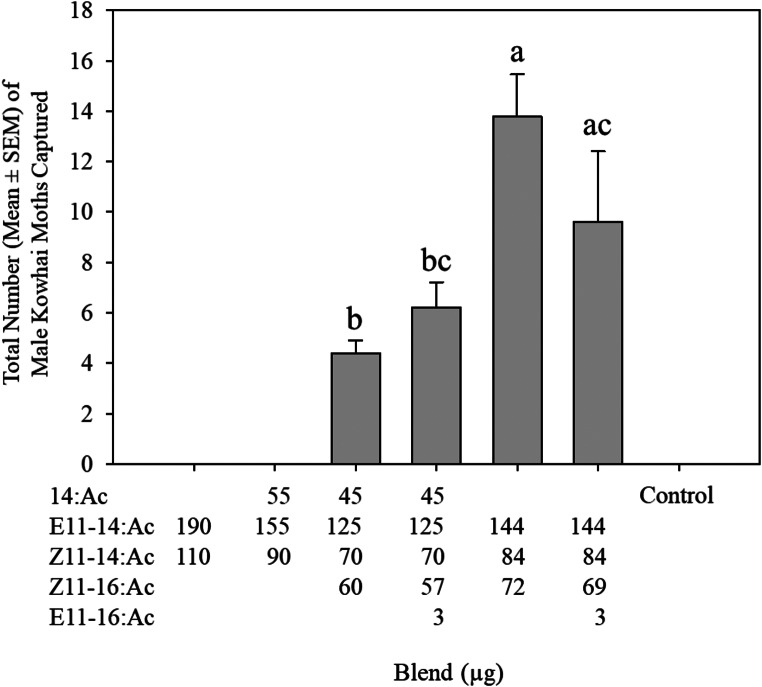
Mean total trap catch ± SE of *Uresiphita polygonalis maorialis* males caught in traps baited with various pheromone blends. Treatments labelled with the same letters are not significantly different *(P* > 0.05)

**Fig. 4:**
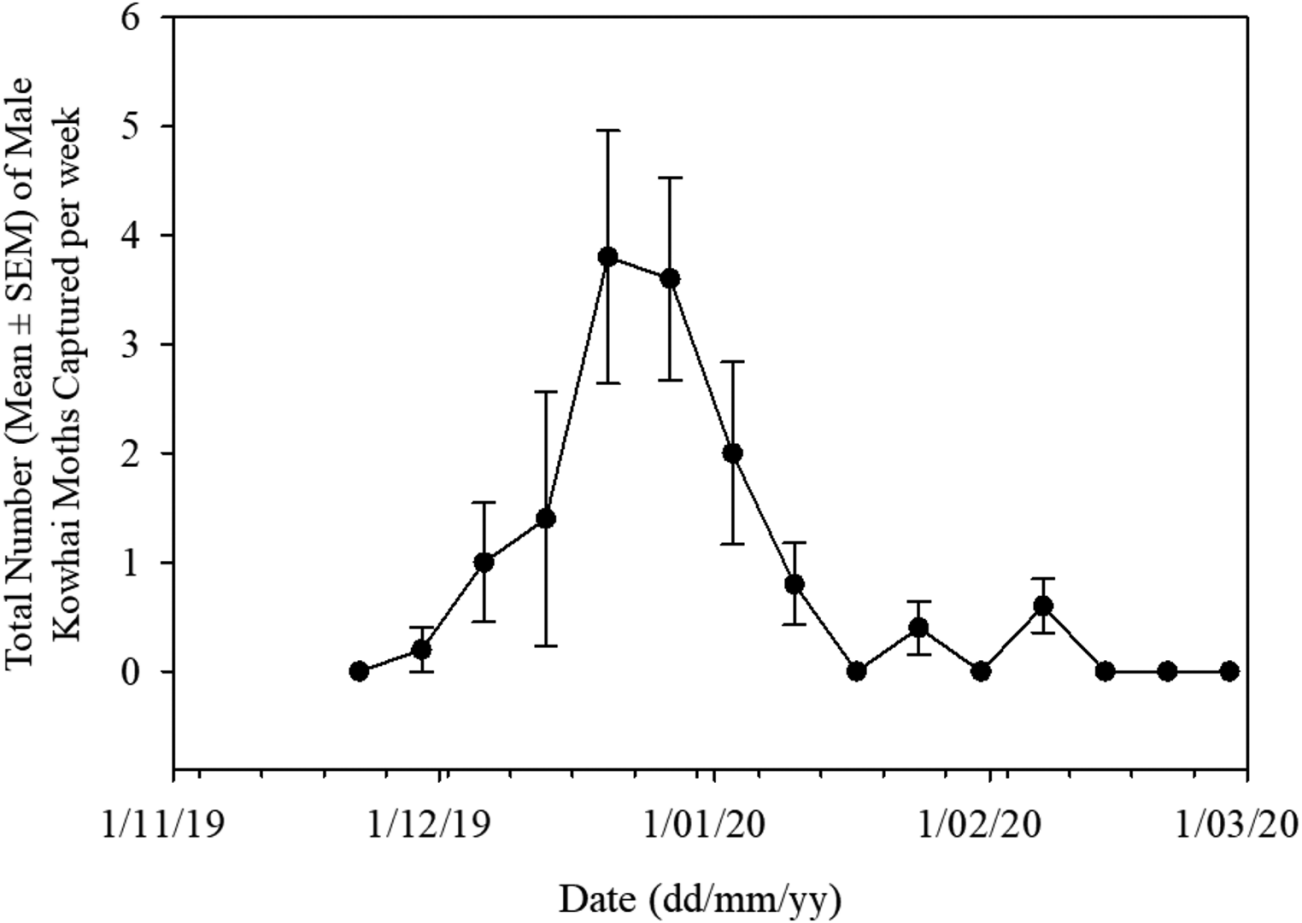
Total number (Mean ± SE) of male *Uresiphita polygonalis maorialis* caught per week over a 15-week period. The sample size for each week was five traps (*N* = 5). The lures used in this experiment contained a three-component blend of E11-14:Ac, Z11-14:Ac, and Z11-16:Ac in amounts of 144 µg, 84 µg, and 72 µg, respectively

This trial primarily aimed at the characterization of the pheromone blend of *U. polygonalis maorialis*. Pheromone blend composition significantly affected the number of males caught in traps (F_3,16_=5.8, *P* < 0.05). No males were caught in traps baited with the binary blend of the two main compounds (E11-14:Ac and Z11-14:Ac) at the ratio present in the sex pheromone gland. Similarly, no males were caught in traps baited with the three-component blend containing the binary blend of E11-14:Ac and Z11-14:Ac plus 14:Ac (Fig. 3). Males were caught in pheromone-baited traps only after the addition of Z11-16:Ac to the binary blend of the two main pheromone compounds. The highest number of males were caught in traps baited with a three-component blend containing E11-14:Ac, Z11-14:Ac, and Z11-16:Ac at an amount of 144, 84, and 72 µg, respectively (Fig. 3). The addition of 1% E11-16:Ac (a compound that did not elicit an EAD response, likely due to its low concentration in the gland extracts) to this blend reduced the number of males caught, but not significantly. However, the simultaneous addition of both 14:Ac and E11-16:Ac significantly reduced the number of males caught (Fig. 3). The results of the mean weekly catch of male *U. polygonalis maorialis* over 15 weeks are provided in Fig. 4. The number of males caught gradually increased, reaching a notable peak in late December. Following this peak, the catch numbers declined, with occasional small increases. Towards the end of the monitoring period, the catch numbers stabilized at 0 moths per trap per week.

#### Testing Three Doses of the three-component Blend

**Fig. 5:**
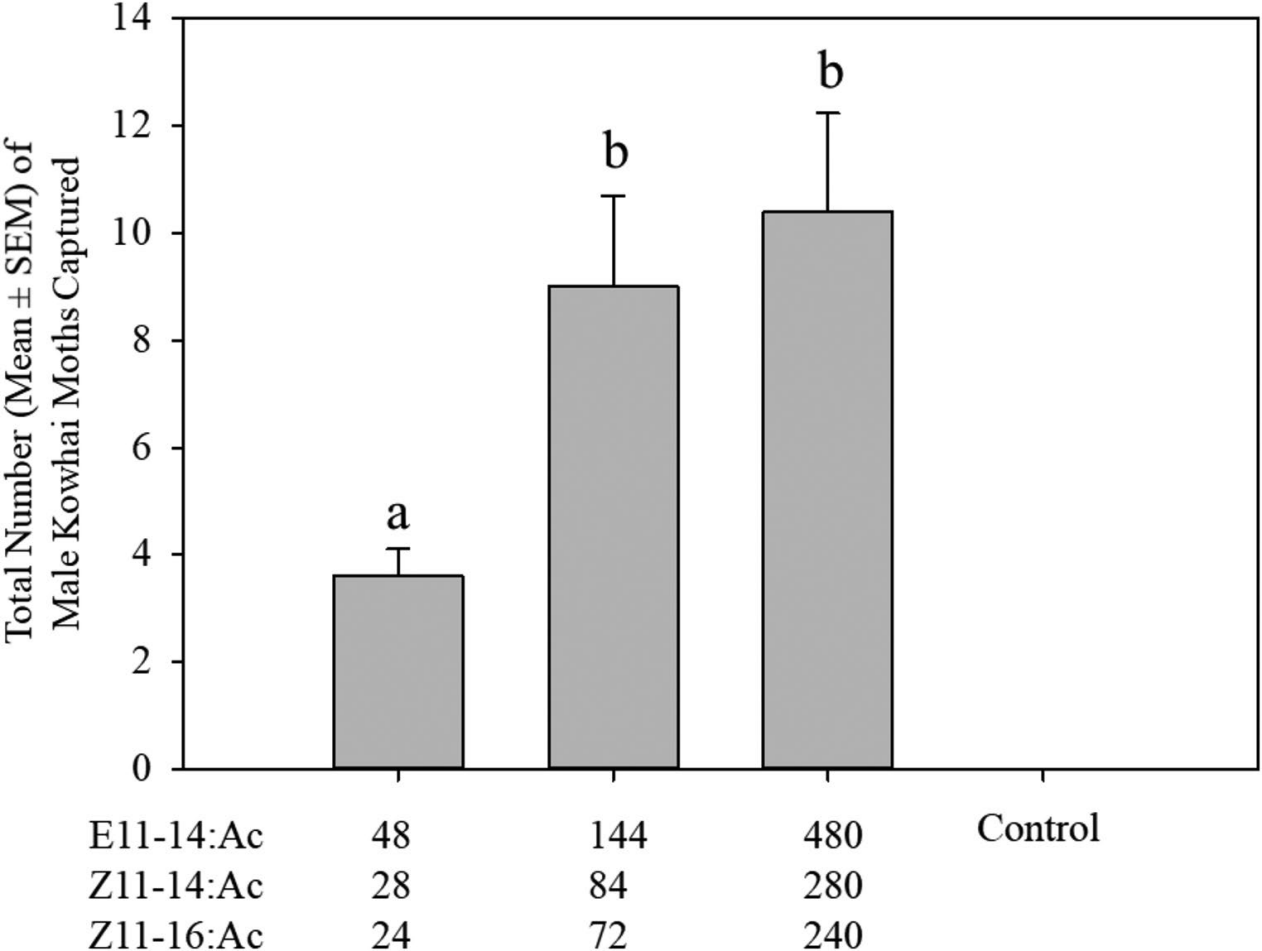
Mean of the total trap catch ± SEM of *Uresiphita polygonalis maorialis* males caught in traps baited with three different doses of the three-component blend (100, 300, and 1000 µg). Treatments labelled with the same letters are not significantly different *(P* > 0.05)

Increasing the dose from 100 µg to 300 µg and 1000 µg led to a significant increase in the number of males caught in the traps (F_2,12_=5.9, *P* < 0.016). The greatest number of males were caught in traps baited with the 1000 µg dose, although this was not significantly different from the catches using the 300 µg dose. The lowest number of males was caught in traps baited with the 100 µg dose (Fig. 5).

## Discussion

The identification of the sex pheromone of *U. polygonalis maorialis* marks a significant advancement in understanding the chemical ecology of this native New Zealand moth. The pheromone blend consisting of E11-14:Ac, Z11-14:Ac, and Z11-16:Ac was found to be the most effective in attracting male moths in field trials. This discovery offers critical insights into the reproductive behavior of this species and presents practical applications for population monitoring and management if needed, especially in the case of an outbreak of this moth.

The results indicate that the two primary pheromone compounds, E11-14:Ac, and Z11-14:Ac are not active when tested alone. Male attraction was observed only when Z11-16:Ac was added to the binary blend of E11-14:Ac, and Z11-14:Ac. The results highlight the importance of multi-component pheromone systems in this native New Zealand species. This finding aligns with previous research on moth pheromone identification in other native to New Zealand species, the pink grass worm, *Tmetolophota atristriga* (El-Sayed and Manning, 2022). A notable example of a crambid moth using similar pheromone compounds (Z11-14:Ac and E11-14:Ac) is the European corn borer, *Ostrinia nubilalis* (Hübner) (Klun et al. 1973). Another example is the perilla leaf pyralid moth, *Pyrausta panopealis* (Walker), which utilizes E11-14:Ac and Z11-14:Ac in its sex pheromone system (Kang and Seol 1995). Many other moth species often involve blends of multiple compounds to achieve optimal attraction (El-Sayed 2024; Millar 2000).

Trace amounts (< 0.3%) of E11-16:Ac were present in the gland extracts, the addition of 1% of this compound to the three-component blend reduced the trap catch although not significantly. However, the simultaneous addition of both 14:Ac and E11-16:Ac resulted in a significant reduction of male attraction. It is possible that the presence of E11-16:Ac in the extracts is due to the degradation of Z11-16:Ac during GC-MS analysis, while 14:Ac might act as a precursor compound in the pheromone biosynthesis pathway. This suggests that these two compounds are not part of the sex pheromone blend of this species. The inhibitory effect observed could be due to these two compounds being utilized by closely related species thus playing a role of reproductive isolation. A similar effect was observed in the codling moth, *Cydia pomonella*, (L), where the analysis of sex pheromone extracts using GC-MS led to the degradation of codlemone and the formation of trace amounts of three geometric isomers (El-Sayed et al., 1998). These isomers were found to inhibit male attraction to codlemone (El-Sayed et al. 2021). The identification of E11-14:Ac, Z11-14:Ac, and Z11-16:Ac in the pheromone gland extracts is consistent with the chemical profiles of pheromones in other crambid species (El-Sayed 2024; Toth et al. 2012; Foster and Harris 1997). Although native to New Zealand, the chemical structures and chain lengths of these compounds are typical of moth pheromones, suggesting conserved biochemical pathways in pheromone production among related species despite the isolation of New Zealand from the rest of the world.

According to The Pherobase (El-Sayed 2024), the sex pheromones have been identified for 130 species within the Crambidae family, spanning 72 genera. The identification of the sex pheromone for *U. polygonalis maorialis* is particularly noteworthy as it represents the first documented pheromone system for this genus. Among Crambid species, (Z)-11-Hexadecenal is commonly the most prominent compound, followed by E11-14:Ac, Z11-14:Ac, and Z11-16:Ac. The sex pheromone blend discovered for *U. polygonalis maorialis* aligns with these trends. This alignment underscores the evolutionary consistency in pheromone systems across diverse environments within the Crambidae family.

Field trials demonstrated that the blend of E11-14:Ac, Z11-14:Ac, and Z11-16:Ac in ratios similar to those found in the female sex pheromone gland is effective at attracting male *U. polygonalis maorialis*. Further testing of different ratios is necessary to confirm whether this blend represents the optimal ratio for attraction. The enhanced trap catches with this blend compared to other blends emphasize the synergistic effects of multi-component pheromones. This multi-component pheromone is well-documented in pheromone research, where specific combinations of compounds often result in the species-specific chemical signal (Cardé and Minks 1995; Löfstedt 1993).

The trapping data also provide valuable insights into the population dynamics of *U. polygonalis maorialis*. The univoltine pattern observed in Canterbury suggests a single generation per year, which has implications for the timing of pheromone-based monitoring and control strategies. Understanding the seasonal flight patterns of this moth can inform the development of integrated pest management (IPM) programs, particularly in areas where this species may pose a threat to native vegetation or agricultural crops.

The identification of the sex pheromone of *U. polygonalis maorialis* not only contributes to the ecological knowledge of this species but also offers practical benefits for its monitoring and management. The use of pheromone traps can aid in detecting and quantifying population densities, assessing distribution ranges, and implementing targeted control measures. Furthermore, this research adds to the growing body of knowledge on moth pheromones in New Zealand, where native species have been relatively understudied compared to agricultural pests.

Future research should focus on further refining the pheromone blend and exploring its applications in different ecological contexts. In conclusion, the identification of the sex pheromone of *U. polygonalis maorialis* represents a significant step forward in the study of New Zealand’s native moths. The findings have important implications for the ecological understanding and management of this species, highlighting the value of pheromone research in both basic and applied entomology.

## Electronic Supplementary Material

Below is the link to the electronic supplementary material.

Supplementary Material 1

## Data Availability

No datasets were generated or analysed during the current study.
