# Supplementary figures and images for: Identification of Components of the Sex Pheromone of the kōwhai Moth, Uresiphita Polygonalis Maorialis, a New Zealand Native Crambid
Source: J Chem Ecol. 2025 Jan 22;51(1):4. doi: 10.1007/s10886-025-01564-8 (PMC11754372; doi:10.1007/s10886-025-01564-8)

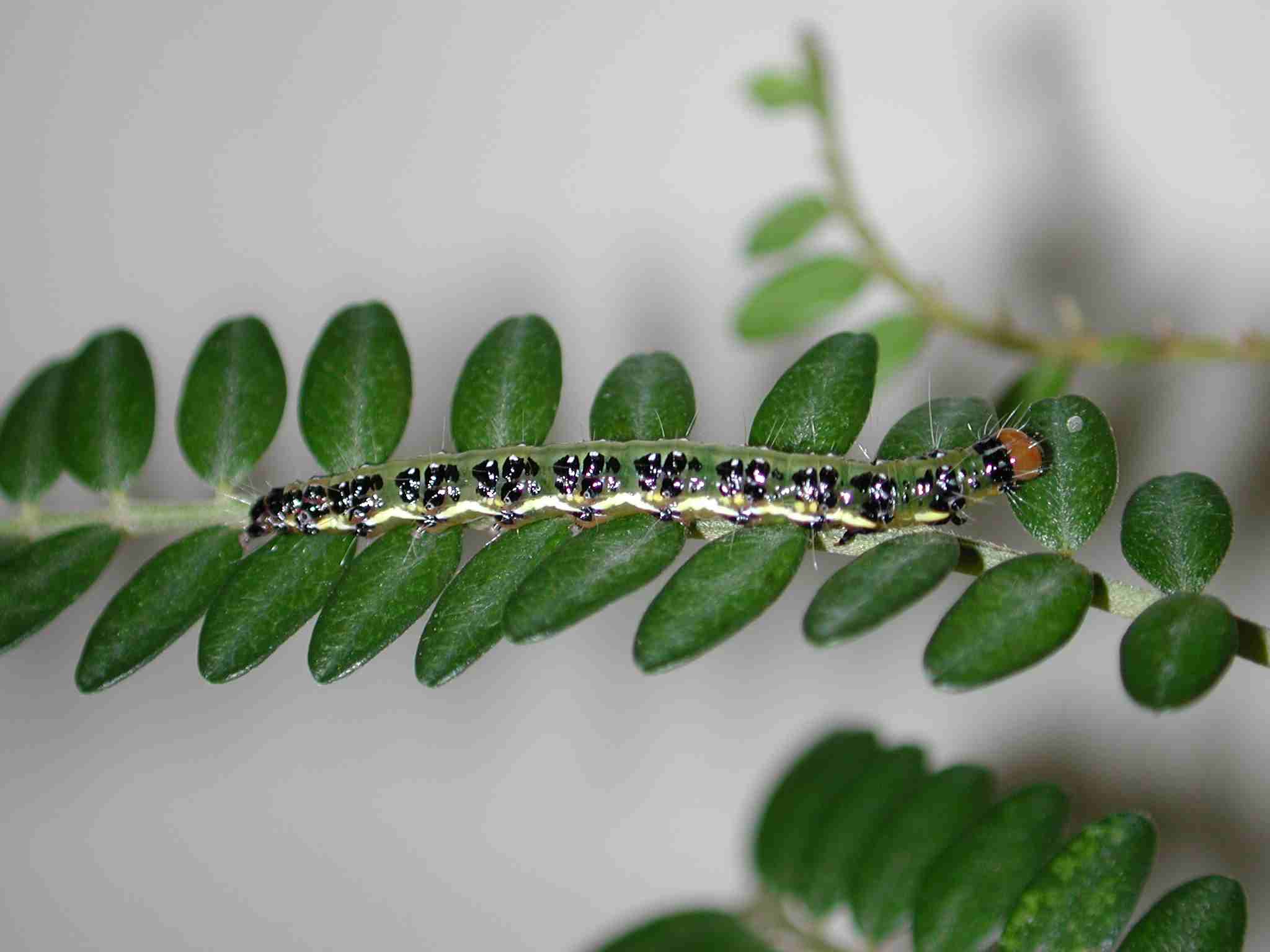

Supplement: Supplementary file 1 — Supplementary Material 1 [file 10886_2025_1564_MOESM1_ESM.jpg]
